# Predominance of the heterozygous CCR5 delta‐24 deletion in African individuals resistant to HIV infection might be related to a defect in CCR5 addressing at the cell surface

**DOI:** 10.1002/jia2.25384

**Published:** 2019-09-04

**Authors:** Vic Arendt, Mathieu Amand, Gilles Iserentant, Morgane Lemaire, Cécile Masquelier, Gilles F Ndayisaba, Chris Verhofstede, Etienne Karita, Susan Allen, Andy Chevigné, Jean‐Claude Schmit, Danielle Perez Bercoff, Carole Seguin‐Devaux

**Affiliations:** ^1^ Department of Infection and Immunity Luxembourg Institute of Health Esch‐sur‐Alzette Luxembourg; ^2^ Centre Hospitalier de Luxembourg National Service of Infectious Diseases Luxembourg Luxembourg; ^3^ Luxembourg (Lux‐Development) Kigali Rwanda; ^4^ Department of Clinical Chemistry, Microbiology and Immunology AIDS Reference Laboratory Ghent University Ghent Belgium; ^5^ Department of Pathology and Laboratory Medicine Emory University School of Medicine Atlanta GA USA

**Keywords:** CCR5, HIV‐1, Africa, mutation, receptor expression, AIDS

## Abstract

**Introduction:**

The chemokine receptor CCR5 is the main co‐receptor for R5‐tropic HIV‐1 variants. We have previously described a novel 24‐base pair deletion in the coding region of CCR5 among individuals from Rwanda. Here, we investigated the prevalence of hCCR5Δ24 in different cohorts and its impact on CCR5 expression and HIV‐1 infection *in vitro*.

**Methods:**

We screened hCCR5Δ24 in a total of 3232 individuals which were either HIV‐1 uninfected, high‐risk HIV‐1 seronegative and seropositive partners from serodiscordant couples, Long‐Term Survivors, or HIV‐1 infected volunteers from Africa (Rwanda, Kenya, Guinea‐Conakry) and Luxembourg, using a real‐time PCR assay. The role of the 24‐base pair deletion on CCR5 expression and HIV infection was assessed in cell lines and PBMC using mRNA quantification, confocal analysis, flow and imaging cytometry.

**Results and Discussion:**

Among the 1661 patients from Rwanda, 12 individuals were heterozygous for hCCR5Δ24 but none were homozygous. Although heterozygosity for this allele may not confer complete resistance to HIV‐1 infection, the prevalence of the mutation was 2.41% (95%CI: 0.43; 8.37) in 83 Long‐Term Survivors (LTS) and 0.99% (95%CI: 0.45; 2.14) in 613 HIV‐1 exposed seronegative members as compared with 0.35% (95% Cl: 0.06; 1.25) in 579 HIV‐1 seropositive members. The prevalence of hCCR5Δ24 was 0.55% (95%CI: 0.15; 1.69) in 547 infants from Kenya but the mutation was not detected in 224 infants from Guinea‐Conakry nor in 800 Caucasian individuals from Luxembourg. Expression of hCCR5Δ24 in cell lines and PBMC showed that the hCCR5Δ24 protein is stably expressed but is not transported to the plasma membrane due to a conformational change. Instead, the mutant receptor was retained intracellularly, colocalized with an endoplasmic reticulum marker and did not mediate HIV‐1 infection. Co‐transfection of hCCR5Δ24 and wtCCR5 did not indicate a transdominant negative effect of CCR5Δ24 on wtCCR5.

**Conclusions:**

Our findings indicate that hCCR5Δ24 is not expressed at the cell surface. This could explain the higher prevalence of the heterozygous hCCR5Δ24 in LTS and HIV‐1 exposed seronegative members from serodiscordant couples. Our data suggest an East‐African localization of this deletion, which needs to be confirmed in larger cohorts from African and non‐African countries.

## 1 Introduction

**Figure 1:**
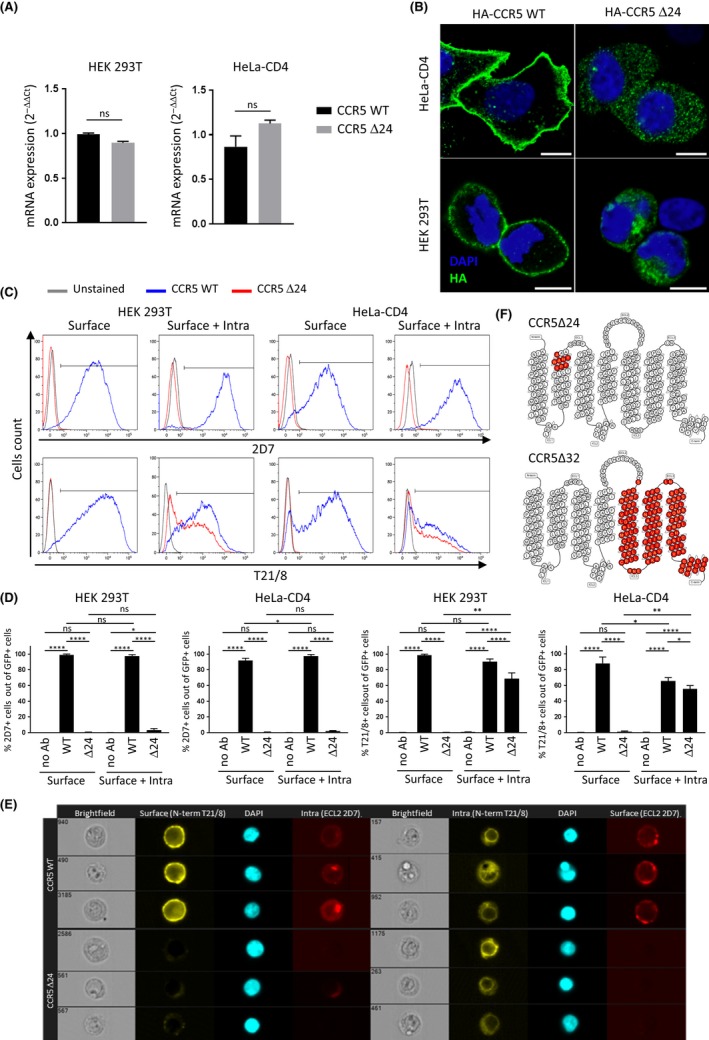
hCCR5Δ24 mutant is not expressed at the cell surface in cell lines. (**A**) The hCCR5Δ24 deletion does not affect the relative mRNA expression levels of the CCR5 receptor in HEK 293T and HeLa‐CD4 cells transiently transfected with pCMV5/HA‐wtCCR5 and pCMV5/HA‐hCCR5Δ24 and measured by qRT‐PCR. GAPDH was used as a reference gene. (**B**) Confocal immunofluorescence analysis reveals hCCR5Δ24 mutant accumulates in the intracellular compartment but not at the cell surface. CCR5 was stained using anti‐HA mAb and the nucleus was stained using DAPI. 10 μm scale. (**C** and **D**) wtCCR5 and hCCR5Δ24 surface or surface +intracellular expression measured by flow cytometry using Ab targeting the NH2‐terminal region or ECL2. (**D**) A GFP reporter vector was added to each transfection in order to analyse CCR5 expression in transfected populations. (**E**) Intracellular hCCR5Δ24 is detectable in transfected HEK 293T cells by imaging cytometry using an Ab targeting the NH2‐terminal region but not ECL2. CCR5 surface or surface + intracellular expression were measured using anti‐CCR5 2D7 (ECL 2) and anti‐CCR5 T21/8 (N‐term) mAbs (**C**,** D** and **E**). Statistical significance was considered when *p *≤* *0.05 (****p ≤* *0.0001, ***p ≤* *0.001, **p ≤* *0.01, *p ≤* *0.05; N = 3 independent experiments). Error bars denote mean ± SD. (**F**) Snake diagram depicting CCR5 topology and identifying the positions occupied by missing residues (red) in the delta 24 (upper panel) and delta 32 (lower level) receptors respectively.

Infection with the Human Immunodeficiency Virus‐1 (HIV‐1) requires the sequential binding of the envelope glycoprotein gp120 to the primary receptor CD4 and a chemokine co‐receptor like CCR5 or CXCR4 which induces conformational changes promoting membrane fusion 1, 2. CCR5 is the main co‐receptor for the R5‐tropic HIV‐1 variants that are most commonly transmitted and predominate during early stages of infection in contrast to the X4 tropic viruses that generally appear in the advanced stage of infection 3, 4, 5. A variant of CCR5 containing a deletional mutation of 32‐base pairs impairs cell surface expression of the co‐receptor due to an early termination of translation (Figure 1). Homozygous individuals for the CCR5Δ32 alleles are highly protected from HIV infection 6, 7, 8. Although heterozygous individuals are susceptible to HIV‐1 infection, their rate of disease progression is usually slower and long‐term survival enhanced, in accordance with a decreased CCR5 expression at the target cell surface 8, 9, 10, 11, 12, 13. The mutant CCR5Δ32 was proposed to heterodimerize with wild‐type CCR5 and hence causes its intracellular retention through a transdominant negative effect 14, 15, 16. Diminished levels of wtCCR5 in CCR5Δ32 heterozygotes were also proposed to result from a simple effect of gene dosage 17.

Accordingly, the CCR5 inhibitor Maraviroc is used in clinical practice against HIV‐1 entry 18, whereas the blocking monoclonal antibody PRO 140 against CCR5 is currently in phase III trial 19. The critical role of CCR5 in HIV infection was emphasized by the haematopoietic stem cell transplantation from CCRΔ32 homozygous donors to HIV‐infected receivers diagnosed with acute myeloid leukaemia or Hodgkin's lymphoma. This resulted in the complete clearance of the virus, and the so called “Berlin” and “London” patients are the only cases of HIV cure to date 20, 21. Several limitations, such as allogenic stem cell transplantation side‐effects or rapid rebound of pre‐existing X4 tropic virus hampered its successful repeat 22, 23, 24, 25. Nevertheless, this discovery stimulated intense research for CCR5 targeted therapies and strategies to downregulate CCR5 expression in order to inhibit HIV infection 26, 27.

In addition to CCR5Δ32, several other CCR5 variants have been described 28, 29, 30. Some of them were characterized by major alterations in their functional response to chemokines or in their potential to modulate HIV‐1 disease progression 6, 31, 32, 33, 34. In Kigali, Rwanda, we identified four individuals heterozygous for a novel 24‐base pair deletion in the coding region of CCR5: an HIV‐infected mother and her child and two individuals from a serodiscordant couple 35. The 24‐base pair deletion involves nucleotides 61,730 to 61,753, encoding 8 amino acids located at the top of the second extracellular transmembrane helix (TM2) (V83–A90) 35 (Figure 1), and partially overlaps the TXP motif of the chemokine receptor which is implicated in chemokine‐induced receptor activation, cell surface expression and HIV envelope‐dependent fusion 36, 37, 38, 39.

The aim of the present study was to investigate the prevalence of hCCR5Δ24 in different cohorts from Rwanda, Kenya, Guinea‐Conakry and Luxembourg to determine whether the deletion can confer resistance to HIV‐1 infection as well as to decipher its geographical localization. The impact of the deletion on CCR5 expression and HIV‐1 infection was further assessed *in vitro*.

## 2 Methods

### 2.1 Study population

Approval of the national ethical committees was obtained and all participants signed an informed consent to participate in this study in Rwanda, Kenya and Luxembourg. From Rwanda, buccal cells were collected on swabs between January 2008 and May 2009 from three different cohorts: 386 uninfected individuals (general population, healthy donors at least 18 years old recruited at the National Reference Hospital of Kigali), 83 Long‐term Survivors (LTS) from a prospective cohort study on the natural history of HIV infection among adult women. These women had been identified as HIV positive in 1986‐1989, when antiretroviral treatment was not available. At the time of enrolment, all women were in clinical stages I/II for more than 10 years 40, 579 HIV positive and 613 HIV‐negative members from a cohort of serodiscordant couples 41. Eligibility criteria of the former cohort included having HIV discordant results (man HIV positive/woman HIV negative or man HIV negative/woman HIV positive), cohabiting and living in Kigali for at least 12 months, age at recruitment between 21 and 65 years for men, and 21 to 45 years for women. Both discordant couples and LTS were monitored in their respective cohorts every three months, and the recruitment of participants into this study in one single visit coincided with one follow‐up visit in the discordant/LTS studies. From Kenya, 547 anonymized whole blood samples of new‐borns collected between 1998 and 2000 in Mombasa from a mother‐to‐child HIV‐1 transmission cohort were included 42. The HIV‐positive mothers were recruited in Coast Provincial General Hospital, in the non‐intervention arm of a study evaluating the effect of vaginal lavage with diluted chlorhexidine on MTCT. All women in active labour, at least 18 years old and living within a reasonable distance to allow follow‐up were eligible. Women admitted in second stage, with obstetric complications, undergoing caesarean section or delivering a stillborn were excluded. From Guinea‐Conakry, anonymized Dried Blood Spots (DBS) from infants born from any HIV‐infected mother attending the clinic of Médecins Sans Frontières (MSF) in Conakry were shipped to Luxembourg for HIV diagnostic. From Luxembourg, buffy coats of 800 Caucasian adults (500 healthy HIV‐1 seronegative individuals or health care workers and 300 HIV‐1 infected adults of the Luxembourg HIV cohort collected at the Centre Hospitalier de Luxembourg between 1992 and 2000) 43 were included.

### 2.2 Screening of hCCR5Δ24

DNA was extracted from buccal swabs, buffy coat or whole blood using the NucliSENS^®^ easyMAG™ system (bioMérieux SA, Marcy l'Etoile, France) according to the manufacturer's recommendations. DNA was extracted from DBS using Chelex resin 44. Screening for the hCCR5Δ24 deletion was performed by allelic discrimination using a custom TaqMan assay on the ABI 7500 Fast Real Time System (Applied Biosystems, Brussels, Belgium). The TaqMan genotyping assay contained a sense (5′‐GCC‐ATC‐TCT‐GAC‐CTG‐TTT‐TTC‐C‐3′) and antisense (5′‐GCC‐TAT‐AAA‐ATA‐GAG‐CCC‐TGT‐CAA‐GA‐3′) primer, one VIC‐labelled probe for the wild‐type (5′‐CTT‐CTG‐GGC‐TCA‐CTA‐TG‐MGB‐NFQ‐3′) allele and a FAM‐labelled probe matched to the mutant (5′‐TCT‐TAC‐TGC‐CGC‐CCA‐GTG‐MGB‐NFQ‐3′). The probes contained a non‐fluorescent quencher and a minor groove binder (MGB). TaqMan Universal PCR Master Mix was used (Applied Biosystems, Belgium).

### 2.3 Plasmids and cells

Human CCR5 (GenBank:X91492.1) expressed in the pCMV5 vector (Ted Ross, University of Pittsburgh) contains the sequence of the influenza HA‐derived epitope (NH2‐YPYDVPDYA‐COOH) inserted between CCR5 amino acid residues two and three by PCR 45. CCR5 fused to FLAG sequence at its N terminus (NH2‐MDYKDDDDK) was constructed by PCR using the following primers (sense: P‐5′‐AAA‐CTT‐AAG‐CTT‐AAG‐AGG‐TCA‐TTG‐TTC‐ACC‐ATG‐GAT‐TAC‐AAG‐GAT‐GAC‐GAC‐GAT‐AAG‐GAT‐TAT‐CAA‐GTG‐TCA‐AGT‐CCA‐ATC‐TAT‐GAC‐A‐3′, antisense 5′‐ ACG‐GGC‐CCT‐CTA‐GAG‐TCG‐AGC‐CCA‐CTT‐GAG‐TCC‐GTG‐TCA‐CAA‐GCC‐CAC‐AGA‐TAT‐TTC‐CT‐3′). The 24‐bp and 32‐bp deletions were inserted into the CCR5 coding region by inverse PCR using the PfuTurbo^®^DNA Polymerase (Stratagene, Amsterdam, The Netherlands) and the following primers (sense: P‐5′‐CCG‐CCC‐AGT‐GGG‐ACT‐TTG‐GAA‐ATA‐3′, antisense 5′‐CAG‐TAA‐GAA‐GGA‐AAA‐ACA‐GGT‐CAG‐AGA‐TG‐3′) and (sense: P‐5′‐ CT‐GCA‐GCT‐CTC‐ATT‐TTC‐CAT‐ACA‐TTA‐AAG‐ATA‐GTC‐ATC‐TTG‐GGG‐C‐3′, antisense 5′‐ GCC‐CCA‐AGA‐TGA‐CTA‐TCT‐TTA‐ATG‐TAT‐GGA‐AAA‐TGA‐GAG‐CTG‐CAG‐3′) respectively. HEK293T (ATCC) and HeLa‐CD4 cells (AIDS Research and Reference Reagent Program, NIAID) were cultured in DMEM containing 10% heat inactivated fetal bovine serum (FBS), 2 mmol/L L‐glutamine, 100 units/mL penicillin, 100 μg/mL streptomycin and for HeLa‐CD4 cells 200 μg/mL geneticin (Invitrogen, Merelbeke, Belgium). HEK293T and HeLa‐CD4 cells were transfected with DNA (2.5 μg for 1.2 × 10^6^ cells) using JetPRIME (Polyplus). For cotransfections, equimolar amounts of wtCCR5 and hCCR524, hCCR5D32 or empty pcDNA3.1 (mock) were used for a total amount of 2 μg of DNA. A total of 0.5 μg of a pCDNA3.1 GFP expression plasmid was added. Peripheral Blood mononuclear cells (PBMC) were isolated from healthy donors using Ficoll gradient centrifugation and maintained in RPMI complemented with 10% FBS, 2 mmol/L glutamine, 100 units/mL penicillin and 0.1 mg/mL streptomycin. Unstimulated fresh PBMC were transfected with CCR5 or hCCR5Δ24 plasmids using the Nucleofector™ technology from Amaxa Biosytems and the Amaxa Human T cells Nucleofector kit according to the manufacturer's protocols.

### 2.4 Gene expression analysis

Total mRNA from transfected HEK293T cells was extracted using the RNeasy Mini Kit (QIAGEN, Venlo, The Netherlands). RNA concentration was quantified with a Nanodrop Spectrophotometer. The High Capacity cDNA RT kit (Applied Bisosystems) was used for RT‐PCR with CCR5Δ24 Primers (CCR5Δ24 sense :5′‐AAG‐GTC‐TTC‐ATT‐ACA‐CCT‐GCA‐GC‐3′; CCR5Δ24 antisense :5′‐AGC‐AGC‐GGC‐AGG‐ACC‐A‐3′) and a TAMRA Probe (hCCR5Δ32WT: 5′‐FAM‐ACA‐GTC‐AGT‐ATC‐AAT‐TCT‐GGA‐AGA‐ATT‐TCC‐AG‐TAMRA‐3′). wtCCR5 and hCCR5Δ24 relative quantification values were measured in triplicate and calculated according to the 2^−ΔΔCt^ method using the GADPH housekeeping gene for normalization and values from one healthy control donor.

### 2.5 Flow cytometry and imagestream analyses

For co‐transfections with wtCCR5 and hCCR5Δ24 or hCCR5Δ32 mutants, a mouse anti‐HA tag monoclonal mAb (HA.C5; Abcam, Cambridge, UK) was revealed with a secondary goat anti‐mouse PE‐conjugated Ab for the detection of hCCR5Δ24 and hCCR5Δ32 N‐terminal HA‐tagged constructs. The N‐terminal Flag‐tagged wtCCR5 construct was detected using a goat anti‐ECS (DDDDK) primary Ab (Bethyl Laboratories, Antwerp, Belgium) in combination with a donkey anti‐goat Alexa Fluor 647‐conjugated Ab. PE‐conjugated anti‐CCR5 mAb T21/8 clone (eBioscience, Vienna, Austria) was used to stain the N‐terminal region of the receptor, whereas APC‐conjugated anti‐CCR5 mAb 2D7 clone (Becton Dickinson, Franklin Lakes, NJ, USA) recognizes the second extracellular loop (ECL2) of CCR5. For conventional flow cytometry experiments, cells were first stained with LIVE/DEAD Fixable Near‐IR Dead cell stain kit (Thermo Fisher Scientific, Merelbeke, Belgium) and then fixed for 20 minutes with 1% paraformaldehyde. Staining of cells in FACS buffer or in 1X Permeabilization buffer (eBioscience) was performed to achieve either surface staining or surface + intracellular staining respectively. For PBMC staining conjugated PE or APC mouse anti‐human CD4 RPA‐T4 clone (Becton Dickinson, USA) was added to gate on CD4^+^ T cells. For ImageStream cytometry, cells were stained using BD Cytofix/Cytoperm™ (Becton Dickinson, USA) according to the manufacturer's instructions and by adding DAPI for nucleus visualization. Acquisition was performed using the BD LSR Fortessa SORP Cytometer (Becton Dickinson, USA) and for ImageStream, single cells were selected by Gradient RMS_M01, Area_M01 versus Aspect Ratio_M01 and intensity_M12 (ImageStreamX, Amnis Corporation, Seattle, WA, USA).

### 2.6 Confocal microscopy analysis

Cells were fixed with 4% paraformaldehyde (Sigma‐Aldrich, Overijse, Belgium) during 20 minutes at 4°C. A double staining was performed with rabbit anti‐HA Tag antibody (Sigma, Belgium), anti‐CCR5 purified mouse (Becton Dickinson, USA), anti‐Golgi mouse monoclonal to 58k and an anti‐endoplasmic reticulum mouse monoclonal to PDI (Abcam). For intracellular staining 0.1% of Triton 100X (Sigma) was added to the blocking buffer. Staining was revealed either with an Alexa Fluor 488‐ conjugated goat anti‐rabbit IgG or an Alexa Fluor 568‐conjugated goat anti‐mouse IgG (both from Invitrogen, Belgium), and DAPI for DNA staining. Confocal fluorescent images were captured using a Zeiss LSM510 META confocal laser scanning microscope (Jena, Germany) equipped with a 40x Plan‐NeoFluar oil immersion objective (numerical aperture 1.3). Confocal images were deconvolved using the Huygens Essential image processing software package (Scientific Volume Imaging, Netherlands). Pearson's colocalization correlation coefficients measuring the overlap of green and red pixel intensities were calculated for regions of interest (ROI) corresponding to anti‐HA‐positive cells using the Colocalization Threshold plugin for Image J.

### 2.7 HIV infection in HeLa cells

Pseudotyped viral particles bearing the HXB2 (X4), ADA (R5) or BaL (R5) HIV Envs were produced as previously described 46. HeLa‐CD4 cells were transfected with wtCCR5, hCCR5Δ24 or hCCR5Δ32 as described above and seeded in 96‐well plates at a density of 40.10^4^ cells/well after 24 hours. Forty‐eight hours post‐transfection, transfected cells (triplicate wells) were infected with 250 ng/mL of pseudotyped virions by spinoculation for 2 hours at 1200 g at 25°C, and then incubated at 37°C for 60 hours. Infection was monitored by measuring Firefly Luciferase in cell lysates.

### 2.8 Statistical analyses

Statistical analyses for the hCCR5Δ24 prevalence in the different cohorts were performed using a Fisher‐T test. The unpaired Mann‐Whitney *t*‐test was used to assess statistical differences between two independent groups for flow cytometry and HIV infection analyses. One‐way analysis of variance (ANOVA) was used to assess statistical differences between multiple groups. Results were considered significant if *p* < 0.05. Results are presented as mean ± SD. Prism software (GraphPad, San Diego, CA) was used to perform statistical analysis. *p ≤ 0.05, **p ≤* *0.01, ***p ≤* *0.001.

## 3 Results and Discussion

We first investigated the prevalence of the hCCR5Δ24 deletion in cohorts of healthy uninfected volunteers, HIV‐infected LTS and HIV‐1 seropositive and HIV‐1 exposed seronegative (ESN) members from serodiscordant couples (Table 1). In Rwanda, among 1661 individuals, 1649 individuals harboured two wild‐type CCR5 alleles (99.27%, 95% CI: 98.74; 99.58) and 12 individuals were heterozygous for hCCR5Δ24, representing a prevalence of 0.73%, (95% CI: 0.42; 1.26) for the deficient allele. Two of 83 LTS were heterozygous for the hCCR5Δ24 allele (2.41%, 95% CI: 0.43; 8.37) compared with six of 613 ESN (0.99%, 95% CI: 0.45; 2.14), two of 579 HIV‐1 seropositive members (0.35%, 95% Cl: 0.06; 1.25), and two of 386 individuals from the general population (0.52%, 95% CI: 0.09; 1.87). The highest prevalence of the hCCR5Δ24 was found among LTS and ESN although these differences did not reach statistical significance compared to HIV‐1 seropositive members (*p *=* *0.078 for LTS and *p *=* *0.288 for ESN), probably because of the limited number of individuals with a deficient allele. None of the individuals were homozygous for hCCR5Δ24. Ultimately, the predominance of hCCR5Δ24 heterozygosity in LTS and ESN indicates that this mutation may mimic hCCR5Δ32 heterozygosity which reduces the rate of disease progression 8, 9, 10, 11, 12, 47, 48 and confers some protection against HIV transmission 49, 50.

**Table 1:** Prevalence of the hCCR5Δ24 in the Rwandese population

Cohort	Number	Heterozygous carriers hCCR5Δ24	Prevalence rate (%) (95% CI)
General population	386	2	0.52 (0.06 to 1.87)
HIV‐1 seropositive members from serodiscordant couples	579	2	0.35 (0.04 to 1.25)
HIV‐1 exposed seronegative members from serodiscordant couples	613	6	0.98 (0.36 to 2.13)^a^
Long‐Term Survivors	83	2	2.41 (0.29‐8.70)^b^

^a^ *p *=* *0.288 HIV‐negative versus HIV‐positive members from serodiscordant couples; ^b^ *p *=* *0.078 LTS versus HIV‐positive members from serodiscordant couples.

We next screened for the hCCR5Δ24 deletion in other African populations and detected it in 3 of 547 infants from Mombasa, Kenya 42, 51 resulting in a prevalence of 0.55% (95% CI: 0.15; 1.69). The deletion was not detected in any of the 224 infants born from seropositive mothers from a MSF programme of Guinea Conakry nor in 500 HIV‐1 seronegative and 300 HIV‐1 seropositive Caucasians from Luxembourg, thus suggesting a specific East African localization.

This frequency distribution contrasts with the frequency distribution of CCR5Δ32, which is high in Caucasians but very low among Africans and Asians 7, 8, 52, 53, 54. On the one hand it has been hypothesized that the distribution of the CCR5Δ32 allele in Europe results from the positive selective pressure attributed by black plague or smallpox 55, 56, 57, 58. On the other hand, hCCR5Δ32 was shown to be enriched in patients with tick‐borne encephalitis 59, 60 and to be linked with increased risk of fatal outcome of West Nile Virus infection 61. Similarly, we might assume that hCCR5Δ24 may have been selected in Africa by other infectious diseases. The homozygous wtCCR5 individuals are indeed more common in Crimean‐Congo haemorrhagic fever infected patients 62 and CCR5 knock‐out mice are resistant to lethal Dengue virus infection 63. In this regard, the putative East African localization of the hCCR5Δ24 is remarkable. Further investigations are needed to clarify whether its peculiar prevalence in East African cohorts but absence in Guinea Conakry (West Africa) and Luxembourg may be related to a negative or positive selective pressure due to increased or decreased susceptibility to infections. Ultimately, Africa is characterized by an extensive ethnic diversity with ancestral population genetic clusters that correlate with self‐described ethnicity, shared cultural and/or linguistic properties 64. This could also explain the geographical localization of the hCCR5Δ24 in East Africa.

Our cohort analysis suggests that hCCR5Δ24 heterozygosity may delay progression to AIDS but the limited size of the cohorts, the low prevalence of the hCCR5Δ24 allele and the absence of homozygous individuals did not provide enough statistical evidence. We therefore used an *in vitro* approach to assess the impact of the CCR5 variant.

**Figure 2:**
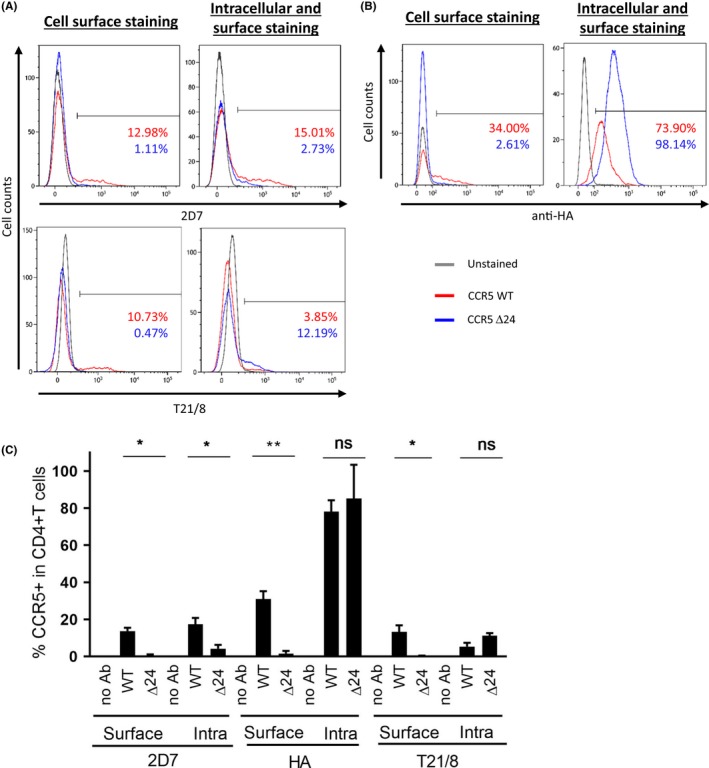
hCCR5Δ24 is not expressed at the cell surface of CD4^+^ T cells in PBMCs.(**A** and **B**). CCR5 surface and intracellular expression was measured by flow cytometry in human CD4^+^ T cells from PBMCs transiently transfected with pCMV5/HA‐wtCCR5 and pCMV5/HA‐hCCR5Δ24 using anti‐CCR5 2D7 (ECL 2) and anti‐CCR5 T21/8 (N‐term) mAbs (**A**) and anti‐HA mAbs (**C**). Statistical analyses of the flow cytometry experiments from (**A**). Statistical significance was considered when *p *≤* *0.05 (**p ≤* *0.01, *p ≤* *0.05; N = 3 independent experiments). Error bars denote mean ± SD.

Homozygous individuals harbouring hCCR5Δ32 are highly resistant to HIV‐1 infection 6, 7, 8 due to a premature truncation of the receptor which abolishes its expression at the cell surface 7, 65 similarly to other natural CCR5 mutants 31. To investigate whether hCCR5Δ24 mutant cell‐surface expression was similarly impaired, we transiently transfected HEK‐293T and HeLa‐CD4 cells with a pCMV5 expression plasmid containing wild‐type hCCR5 or mutant hCCR5Δ24 sequences, both including an HA tag in the N‐terminus of the receptor. Expression levels of wtCCR5 and hCCR5Δ24 mRNA were found to be equivalent in each cell line 24 hours post‐transfection (Figure 1A). We next investigated the receptor cell surface and intracellular expression by confocal microscopy. HA tag staining revealed that the hCCR5Δ24 receptor was not expressed at the cell surface but rather accumulated in the cytoplasm of HEK 293T and HeLa‐CD4 cells (Figure 1B). The intense perinuclear staining suggested that the mutant receptor is efficiently synthesized but retained intracellularly. Impaired cell‐surface expression of the mutant receptor was further confirmed by flow cytometry (Figure 1C,D and Figure S1A). HEK‐293T and HeLa‐CD4 cells were then cotransfected with either hCCR5Δ24 or wtCCR5 and a GFP reporter vector. Flow cytometry was performed using T21/8 mAb and the conformation‐sensitive 2D7 mAb 66 which recognize the N‐terminal region and the second extracellular loop (ECL2) of CCR5 respectively. CCR5 staining was performed at the cell surface alone by fixing and staining cells or at the surface and intracellularly by fixing, permeabilizing and staining cells. CCR5 expression was analysed in GFP‐positive cells to select for the transfected population. We observed that GFP‐negative cells did not express any wt nor mutant CCR5 (data not shown). As shown in Figure 1C,D, hCCR5Δ24 was not detected by any of the mAbs at the cell surface, while the T21/8 mAb targeting the N‐terminal region of hCCR5 revealed similar levels of wtCCR5 and hCCR5Δ24 when the cells were permeabilized. hCCR5Δ24 was not detectable with clone 2D7, which targets ECL2 (Figure 1C,D). These results suggest that the hCCR5Δ24 deletion, located at the top of TM2 close to the disulphide bridge linking ECL1 to ECL2, induced a conformational change in ECL2 67, 68. This conformational change could lead to protein misfolding and may interfere with its surface export, resulting in its intracellular accumulation 69. These observations were confirmed by imaging cytometry (Figure 1E and Figure S1B). hCCR5Δ24 or wtCCR5 expressing HEK293T cells were stained either with T21/8 for surface expression and 2D7 for intracellular and surface expression or T21/8 for intracellular and surface expression and 2D7 for surface expression. Micrographs of three representative cells in each condition clearly indicate that hCCR5Δ24 is only detectable intracellularly using the T21/8 antibody. Similar results were obtained in CD4^+^ T cells from unstimulated human PBMC with the N‐term targeting clone (T21/8) and the conformation‐sensitive 2D7 mAb as well as with a primary anti‐HA tag antibody (Figure 2A) showing the significant absence of surface expression of the mutant protein (Figure 2B).

**Figure 3:**
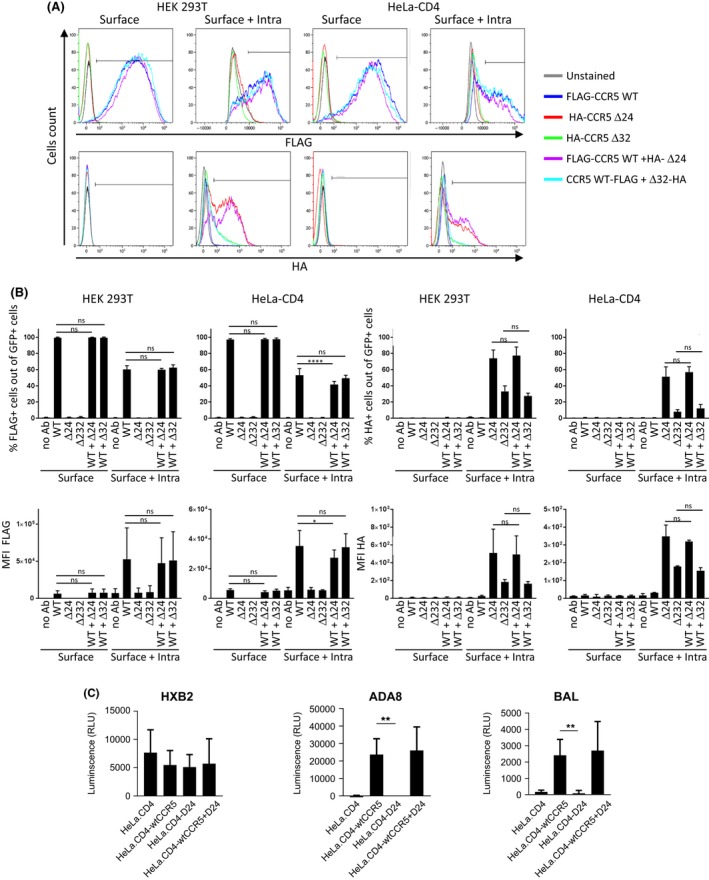
hCCR5Δ24 mutant has no transdominant negative effect on wtCCR5.(**A**) HEK‐293T and HeLa‐CD4 cells were transfected with FLAG‐wtCCR5, HA‐hCCRΔ24 and HA‐hCCR5Δ32 alone or cotransfected with FLAG‐wtCCR5 and HA‐hCCRΔ24 or HA‐hCCR5Δ32 in equimolar ratio. (**A**) GFP reporter vector was added to each transfection in order to analyse CCR5 expression in transfected populations. CCR5 surface or surface + intracellular expression was analysed by flow cytometry using anti‐FLAG and anti‐HA mAbs. (**B**) Quantification of the flow cytometry experiment from A. (**C**) HeLa‐CD4 cells were transfected with FLAG‐wtCCR5, HA‐hCCRΔ24 alone or cotransfected with FLAG‐wtCCR5 and HA‐hCCRΔ24 in equimolar ratios. Transfected cells were infected with HXB2, ADA8 or BaL pseudovirus expressing a Luciferase reporter gene. HIV‐1 infection was quantified by measuring Luciferase‐dependent luminescence. Statistical significance was considered when *p *≤* *0.05 (****p ≤* *0.0001, ***p ≤* *0.001, **p ≤* *0.01, *p ≤* *0.05; N = 3 independent experiments). Error bars denote mean±SD.

We next investigated whether hCCR5Δ24 has a transdominant negative effect on wtCCR5 surface expression. To distinguish between wtCCR5 and the mutated hCCR5Δ24, we inserted a FLAG sequence at the NH2‐terminal end of wtCCR5. FLAG‐wtCCR5 was cotransfected in HEK 293T and HeLa‐CD4 cells with HA‐hCCRΔ24, HA‐hCCR5Δ32 as a control or the empty pcDNA vector in equimolar ratios to mimic co‐expression of wtCCR5 and the mutants. A GFP reporter vector was added to analyse CCR5 expression in transfected populations. CCR5 surface or surface + intracellular expression was analysed by flow cytometry using anti‐FLAG and anti‐HA mAb (Figure 3A,B and Figure S2). wtCCR5 was detected in each condition using the anti‐FLAG mAb, as expected. Again we observed hCCR5Δ24 and hCCR5Δ32 expression using the anti‐HA mAb only when cells were permeabilized prior to staining, but not when only surface expression was evaluated. When FLAG‐wtCCR5 and HA‐hCCRΔ24 were cotransfected, both forms were detectable intracellularly. Importantly their co‐expression did not affect the levels of surface or surface + intracellular wtCCR5 expression, indicating that hCCRΔ24 has no transdominant negative effect on wtCCR5 expression when expressed in an equivalent molar ratio. Similar results were recorded when wtCCR5 was cotransfected with HA‐hCCR5Δ32 (Figure 3A,B).

To examine the impact of hCCRΔ24 on HIV‐1 infection, HeLa‐CD4 cells were transfected with FLAG‐wtCCR5 or HA‐hCCRΔ24 alone, or cotransfected with both FLAG‐wtCCR5‐FLAG + HA‐hCCRΔ24 at a 1:1 ratio. Forty‐eight hours post‐transfection, transfected cells were infected with CCR5 (ADA8 and BaL) or CXCR4 (HXB2) tropic HIV‐1 pseudoviruses. HeLa‐CD4 cells, which naturally express CXCR4 but not CCR5, were infected with HXB2 but not with CCR5 tropic pseudoviruses, as expected. HeLa‐CD4 cells expressing wtCCR5 demonstrated a dramatically enhanced susceptibility to CCR5 tropic infection (Figure 3C). In contrast, hCCRΔ24 expression did not render HeLa‐CD4 cells susceptible to ADA8 or BaL infection. Interestingly HeLa‐CD4 cells cotransfected with wtCCR5 + hCCRΔ24 mediated entry of ADA8 and BaL pseudovirions to levels comparable to HeLa‐CD4‐wtCCR5 cells, thereby confirming the absence of any transdominant negative effect of hCCR5Δ24 or hCCR5Δ32 on wtCCR5.

Our results are in line with previous studies reporting impaired or reduced cell‐surface expression and cytoplasmic retention of several CCR5 mutant proteins such as human CCR5Δ32 7, 65, human CCR5 C101X, FS299 and CCR5‐893(−) mutants 31, 32 or the A73V mutant, which is located in the second transmembrane domain, close to the 24‐base pair deletion 31. A similar effect was reported for sooty and red‐capped mangabeys CCR5Δ24 70, 71, 72 although the 24‐base pair deletion found in CCR5 from African simian species is not located in the same region as human CCR5Δ24.

Levels of CCR5 expression were previously described to be a key determinant in HIV entry 73, 74, 75 and hence pathogenesis 76. Accordingly, we report that the absence of surface expression of hCCR5Δ24 provides a complete protection against HIV‐1 infection, as previously observed for hCCR5Δ32 homozygous individuals 6, 7, 8. In heterozygous wtCCR5/CCR5Δ32 individuals, resistance to HIV transmission and reduced disease progression rate to AIDS were linked to a decrease in CCR5 expression at the target cell surface 8, 9, 10, 11, 12, 49, 50. A transdominant effect of the mutant protein was proposed, resulting in the intracellular sequestration of wtCCR5 14, 15, 16. In our hands, when wtCCR5 + hCCR5Δ24 or wtCCR5 + hCCR5Δ32 were cotransfected using an equivalent molar ratio, the export of the wild‐type protein and target cell infectability by CCR5 tropic HIV‐1 viruses were unaffected. Our results indicate that hCCR5Δ24 and hCCR5Δ32 have no negative transdominant effect in this particular experimental setting aiming at mimicking equivalent gene expression of hCCR5Δ24 and wtCCR5. We also tested cotransfections at a 3:1 molar ratio (for hCCR5Δ24 and wtCCR5 respectively) as previously performed by Benkirane *et al*. 14 who showed transdominant inhibition of CCR5‐mediated HIV‐1 infection by hCCR5Δ32. Although we observed a trend towards decreased wtCCR5 expression when hCCR5Δ24 was cotransfected in large excess, the MFI of wtCCR5 staining in the cotransfection were not significantly different from the wtCCR5 staining when transfected alone in HEK293 T cells and HeLa‐CD4 cells (Figure S3A,B). Furthermore, we did not observe any significant impact of hCCR5Δ24 overexpression on the susceptibility of HeLa‐CD4 cells to infection with CCR5 tropic pseudoviruses (Figure S3C). Altogether, these results indicate that in our experimental setting hCCRΔ24 has no significant transdominant negative effect on wtCCR5. Since Benkirane *et al*. have used a readout that detects viral entry and fusion while our assay detects viral entry only, it is possible that the negative transdominant effect reported by Benkirane *et al*. reflects an impact of mutant CCR5 receptors on cell‐to‐cell fusion mainly, which our assay did not capture. Further experiments would be needed to confirm this hypothesis. Therefore, we propose a simple mechanism of gene dosage rather than a negative transdominant effect in hCCR5Δ32 and hCCR5Δ24 heterozygotes as previously suggested 17. Noteworthily, our experimental setting of co‐transfection may not fully reflect the complex *in vivo* situation found in heterozygous patients.

**Figure 4:**
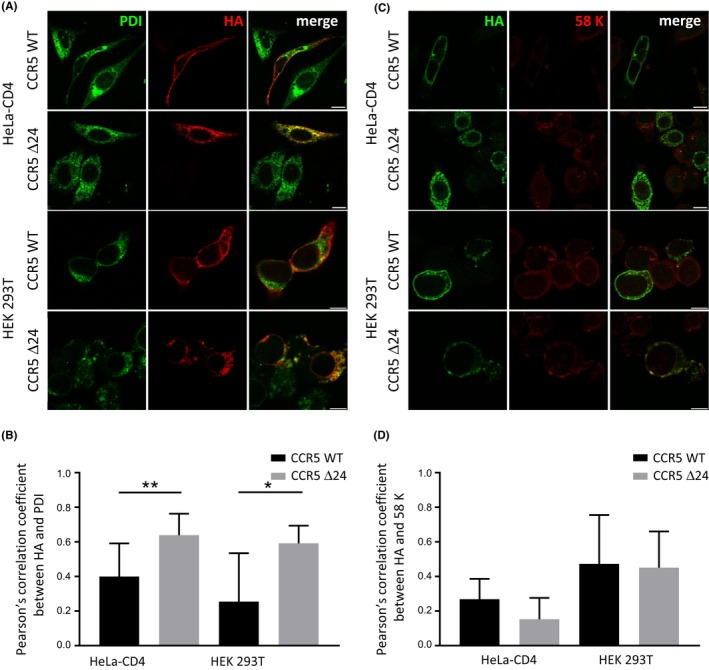
The hCCR5Δ24 mutation increases CCR5 colocalization with Endoplasmic Reticulum but not Golgi markers. HEK 293T and HeLa‐CD4 cells were transiently transfected with pCMV5/HA‐wtCCR5 and pCMV5/HA‐hCCR5Δ24. Representative confocal micrographs of CCR5, ER (**A**) and Golgi (**C**) staining using anti‐HA, anti‐PDI and anti‐58K mAbs respectively. Pearson's colocalization correlation coefficients between anti‐HA and anti‐PDI (**B**) or anti‐58k (**D**) staining were calculated for regions of interest (ROI) corresponding to anti‐HA positive cells using Colocalization Threshold (Image J). Statistical significance was considered when *p *≤* *0.05, **p ≤* *0.01, *p ≤* *0.05; N = 3 independent experiments). Error bars denote mean ± SD. 10 um scale.

The cytoplasmic hCCR5Δ24 accumulation suggests that mutant receptor conformational changes could induce a disruptive intracellular secretory pathway and mutant retention in the Golgi apparatus or the endoplasmic reticulum (ER). To identify the subcellular sites of mutant receptor retention, we immunostained HEK‐293T and HeLa‐CD4 cells transiently expressing HA‐wtCCR5 or HA‐hCCR5Δ24 using an anti‐HA tag Ab with the anti‐58k or anti‐PDI mAbs as Golgi and endoplasmic reticulum (ER) markers respectively (Figure 4A,B). Confocal microscopy revealed that wtCCR5 was systematically distributed at the plasma membrane in both cell lines. In contrast, cells expressing hCCR5Δ24 demonstrated an intracellular staining co‐localizing with the ER marker but not the Golgi marker. These results indicate that conformational changes induced by the hCCR5Δ24 deletion impair CCR5 trafficking, causing its retention into the ER and preventing a correct addressing to the cell surface. Likewise, the CCR5‐893 (‐) and C‐terminal mutants lacking six, five, four or three transmembrane domains or mutated in the basic domain (‐KHIAKRF‐) and the cysteine cluster (‐CKCC‐) were also retained in the ER 14, 17, 77, 78.

## 4 Conclusions

Our results suggest that the hCCR5Δ24 deletion affects ECL1/ECL2 conformation, CCR5 addressing at the cell surface and thus protect from HIV‐1 infection although it has no transdominant negative effect on wtCCR5 expression. These findings might explain the higher prevalence of the heterozygous deletion in Rwandese LTS individuals and in seronegative partners from serodiscordant couples. The small size of our cohorts together with the low prevalence of the mutation may have limited the proper evaluation of hCCR5Δ24 function in HIV infection. We might also speculate that the absence of homozygous hCCR5Δ24 deletion carriers reflects a negative selection due to the high burden of infections in sub‐Saharan Africa. This hypothesis is supported by a recent study showing a deleterious effect of the homozygous hCCR5Δ32 using death register information of 409,693 individuals of British ancestry 78. Analysis of larger cohorts is nevertheless needed to confirm this assumption and the East African localization of the hCCR5Δ24 deletion.

## Competing interest

The authors declare no conflict of interest regarding this work. All authors have submitted the ICMJE Form for disclosure of potential competing interest.

## Authors’ contributions

VA designed and conducted the study in Rwanda and analysed the results. MA analysed experiments, drafted figures and wrote the manuscript. GI performed flow and imaging cytometry experiments and drafted figures. ML performed confocal analysis. CM performed the screening of the deletion and the CCR5 mRNA measurement. GN recruited the participants in Rwanda. CV provided the samples collected in Kenya. EK and SA are managing the LTS and ESS cohorts in Rwanda. DPB and AC constructed the hCCR5Δ24 expression vector and designed the co‐localization and conformational experiments by FACS and confocal microscopy. JCS designed the study and analysed the results. CD designed, supervised the study and wrote the manuscript. All authors participated in the preparation and editing of the manuscript.

## Supporting information


**Figure S1.** hCCR5Δ24 mutant is not expressed at the cell surface of cell lines. (**A**) Representative dot plots of wtCCR5 or hCCR5Δ24 expressing HEK‐293T and HeLa‐CD4 cells stained at the surface or surface + intracellularly with 2D7 and T21/8 mAbs. (**B**) Quantification of the imaging cytometry experiments from Figure 1D. Statistical significance was considered when *p *≤* *0.05 (****p ≤* *0.0001, ***p ≤* *0.001, **p ≤* *0.01, *p ≤* *0.05; N = 3 independent experiments). Error bars denote mean ± SD.

**Figure S2.** hCCR5Δ24 mutant has no transdominant negative effect on wtCCR5 using a 1:1 equimolar ratio. (**A**) Representative dot plots of wtCCR5 or hCCR5Δ24 expressing HEK‐293T and HeLa‐CD4 cells stained at the surface or surface + intracellularly with anti‐FLAG and anti‐HA mAbs.

**Figure S3.** hCCR5Δ24 mutant has no transdominant negative effect on wtCCR5 using a 3:1 ratio. (**A**) HEK‐293T and HeLa‐CD4 cells were transfected with FLAG‐wtCCR5, HA‐hCCRΔ24 and HA‐hCCR5Δ32 alone or cotransfected with FLAG‐wtCCR5 and HA‐hCCRΔ24 or HA‐hCCR5Δ32 at 1:1 or 3:1 ratios. A GFP reporter vector was added to each transfection in order to analyse CCR5 expression in transfected populations. CCR5 surface or surface + intracellular expression was analysed by flow cytometry using anti‐FLAG and anti‐HA mAbs. (**A**) Reports quantification of the flow cytometry experiments. (**B**) HeLa‐CD4 cells were transfected with FLAG‐wtCCR5, HA‐hCCRΔ24 alone or cotransfected with FLAG‐wtCCR5 and HA‐hCCRΔ24 at a 3:1 ratio as in (**A**). (**C**) Transfected cells were infected with HXB2, ADA8 or BaL pseudovirus expressing a Luciferase reporter gene 48 hours post‐transfection. HIV‐1 infection was quantified by measuring Luciferase‐dependent luminescence. Statistical significance was considered when *p *≤* *0.05 (****p ≤* *0.0001, ***p ≤* *0.001, **p ≤* *0.01, *p ≤* *0.05; N = 3 independent experiments). Error bars denote mean ± SD.
