# Supplementary material for: Predominance of the heterozygous CCR5 delta‐24 deletion in African individuals resistant to HIV infection might be related to a defect in CCR5 addressing at the cell surface
Source: J Int AIDS Soc. 2019 Sep 4;22(9):e25384. doi: 10.1002/jia2.25384 (PMC6727025; doi:10.1002/jia2.25384)
Supplement: Supplementary file 1 — Figure S1. hCCR5Δ24 mutant is not expressed at the cell surface of cell lines. (A) Representative dot plots of wtCCR5 or hCCR5Δ24 expressing HEK‐293T and HeLa‐CD4 cells stained at the surface or surface + intracellularly with 2D7 and T21/8 mAbs. (B) Quantification of the imaging cytometry experiments from Figure 1D. Statistical significance was considered when p ≤ 0.05 (****p ≤ 0.0001, ***p ≤ 0.001, **p ≤ 0.01, *p ≤ 0.05; N = 3 independent experiments). Error bars denote mean ± SD. [file JIA2-22-e25384-s001.pptx]

## Slide 1
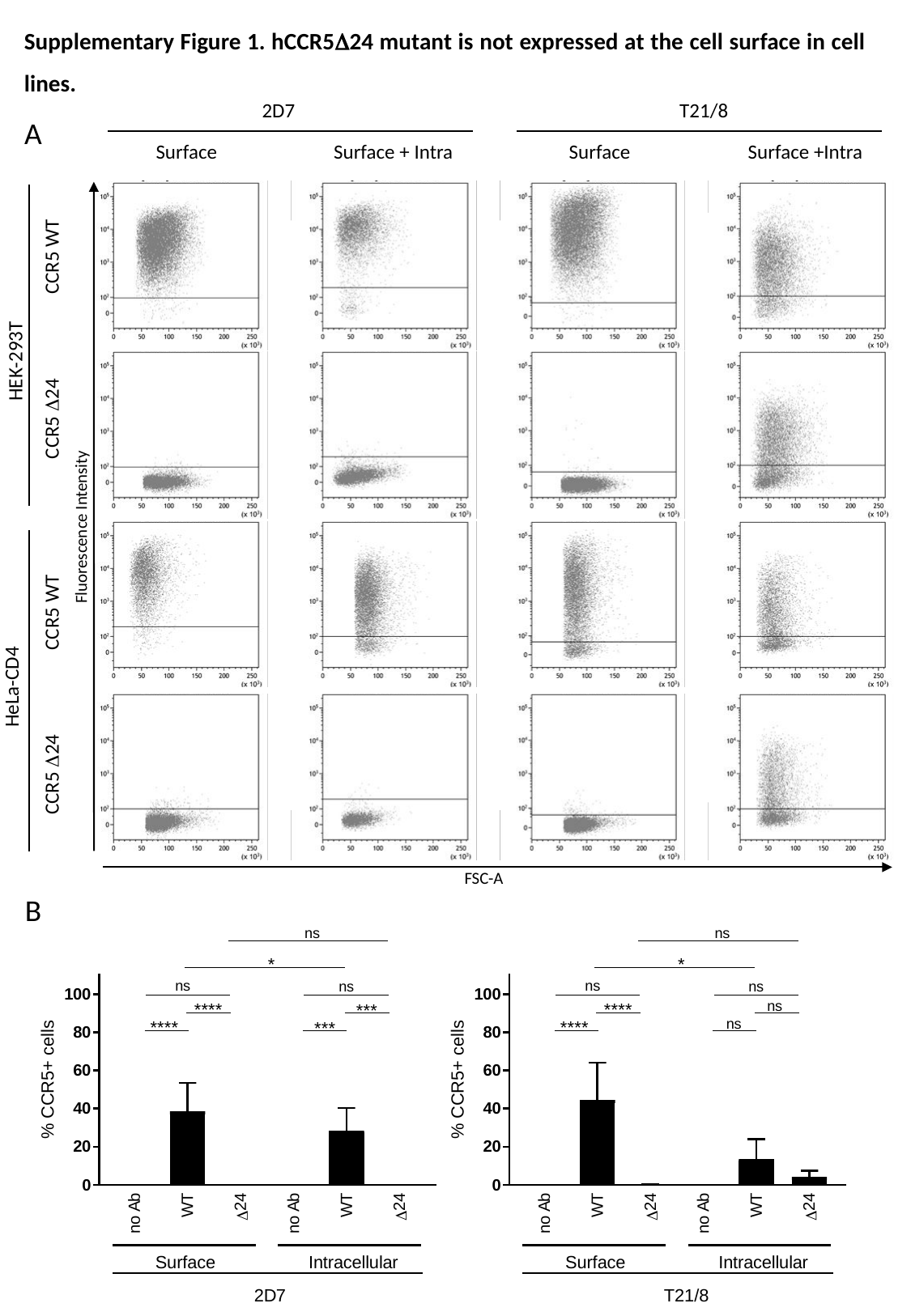

Supplementary Figure 1. hCCR5D24 mutant is not expressed at the cell surface in cell lines.
2D7
T21/8
A
Surface
Surface + Intra
Surface
Surface +Intra
CCR5 WT
HEK-293T
CCR5 D24
Fluorescence Intensity
CCR5 WT
HeLa-CD4
CCR5 D24
FSC-A
B
