# Supplementary material for: Predominance of the heterozygous CCR5 delta‐24 deletion in African individuals resistant to HIV infection might be related to a defect in CCR5 addressing at the cell surface
Source: J Int AIDS Soc. 2019 Sep 4;22(9):e25384. doi: 10.1002/jia2.25384 (PMC6727025; doi:10.1002/jia2.25384)
Supplement: Supplementary file 2 — Figure S2. hCCR5Δ24 mutant has no transdominant negative effect on wtCCR5 using a 1:1 equimolar ratio. (A) Representative dot plots of wtCCR5 or hCCR5Δ24 expressing HEK‐293T and HeLa‐CD4 cells stained at the surface or surface + intracellularly with anti‐FLAG and anti‐HA mAbs. [file JIA2-22-e25384-s002.pptx]

## Slide 1
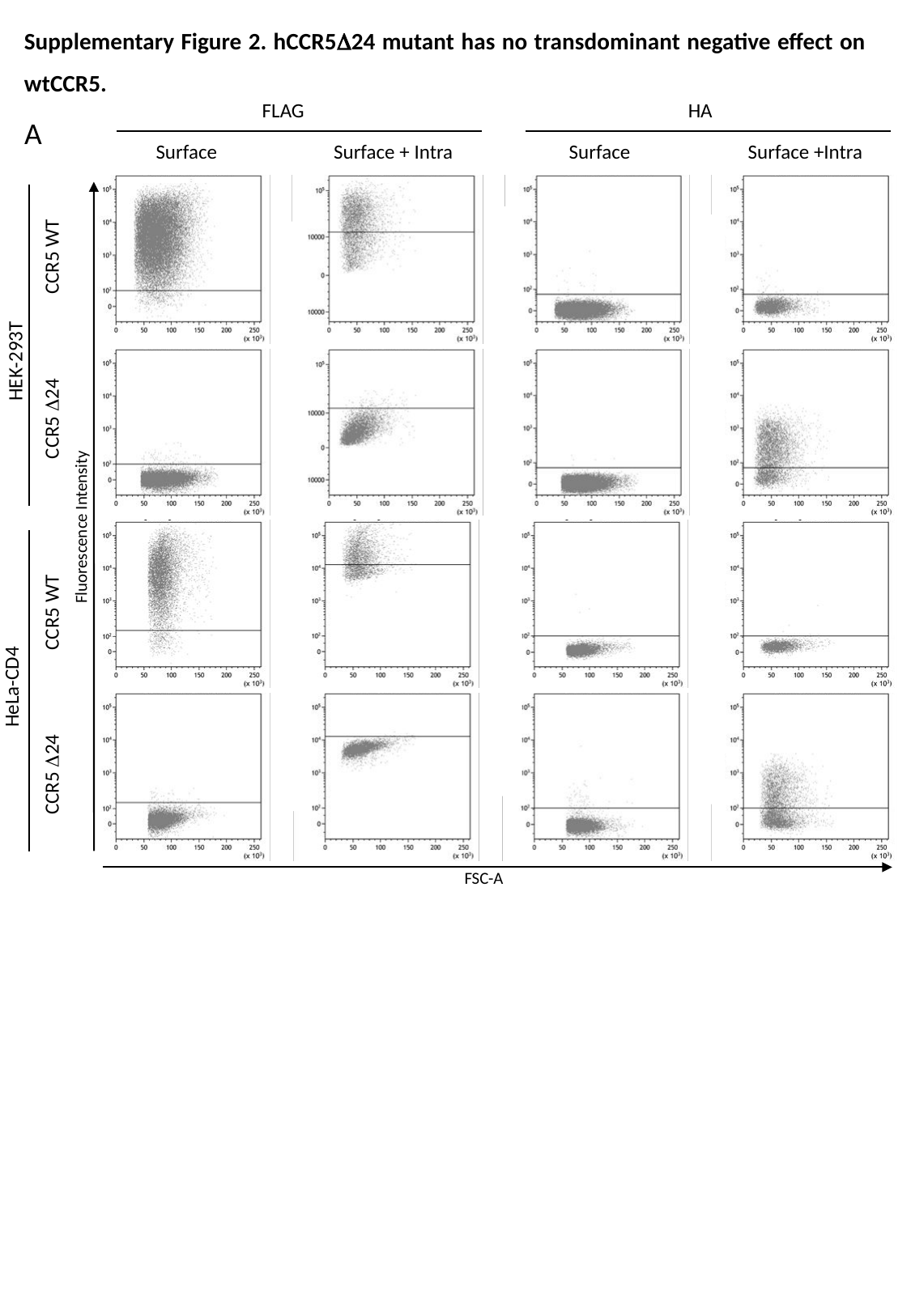

Supplementary Figure 2. hCCR5D24 mutant has no transdominant negative effect on wtCCR5.
FLAG
HA
A
Surface
Surface + Intra
Surface
Surface +Intra
CCR5 WT
HEK-293T
CCR5 D24
Fluorescence Intensity
CCR5 WT
HeLa-CD4
CCR5 D24
FSC-A
