# Supplementary figures and images for: Predominance of the heterozygous CCR5 delta‐24 deletion in African individuals resistant to HIV infection might be related to a defect in CCR5 addressing at the cell surface
Source: J Int AIDS Soc. 2019 Sep 4;22(9):e25384. doi: 10.1002/jia2.25384 (PMC6727025; doi:10.1002/jia2.25384)

## Slide 1
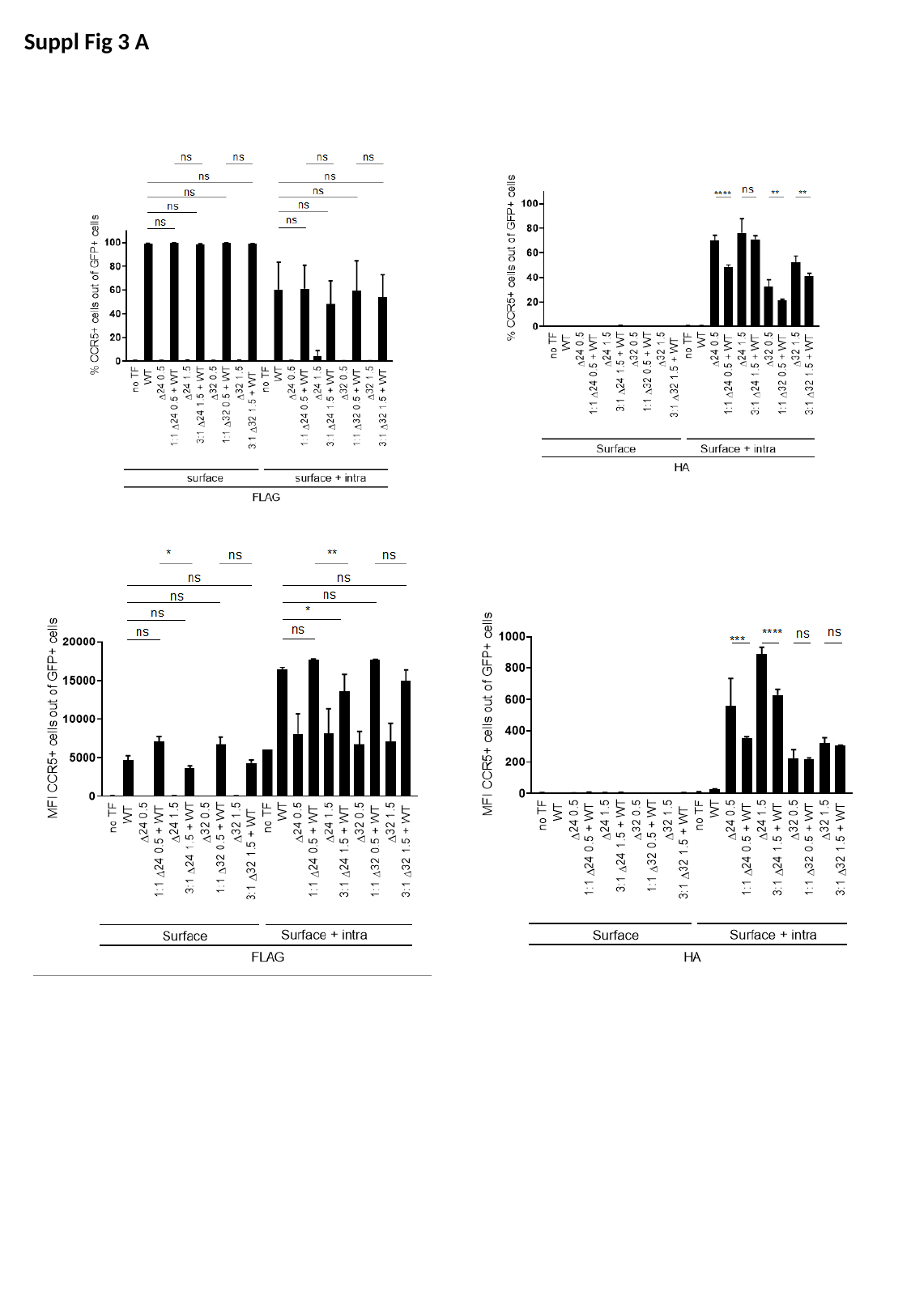

Suppl Fig 3 A

## Slide 2
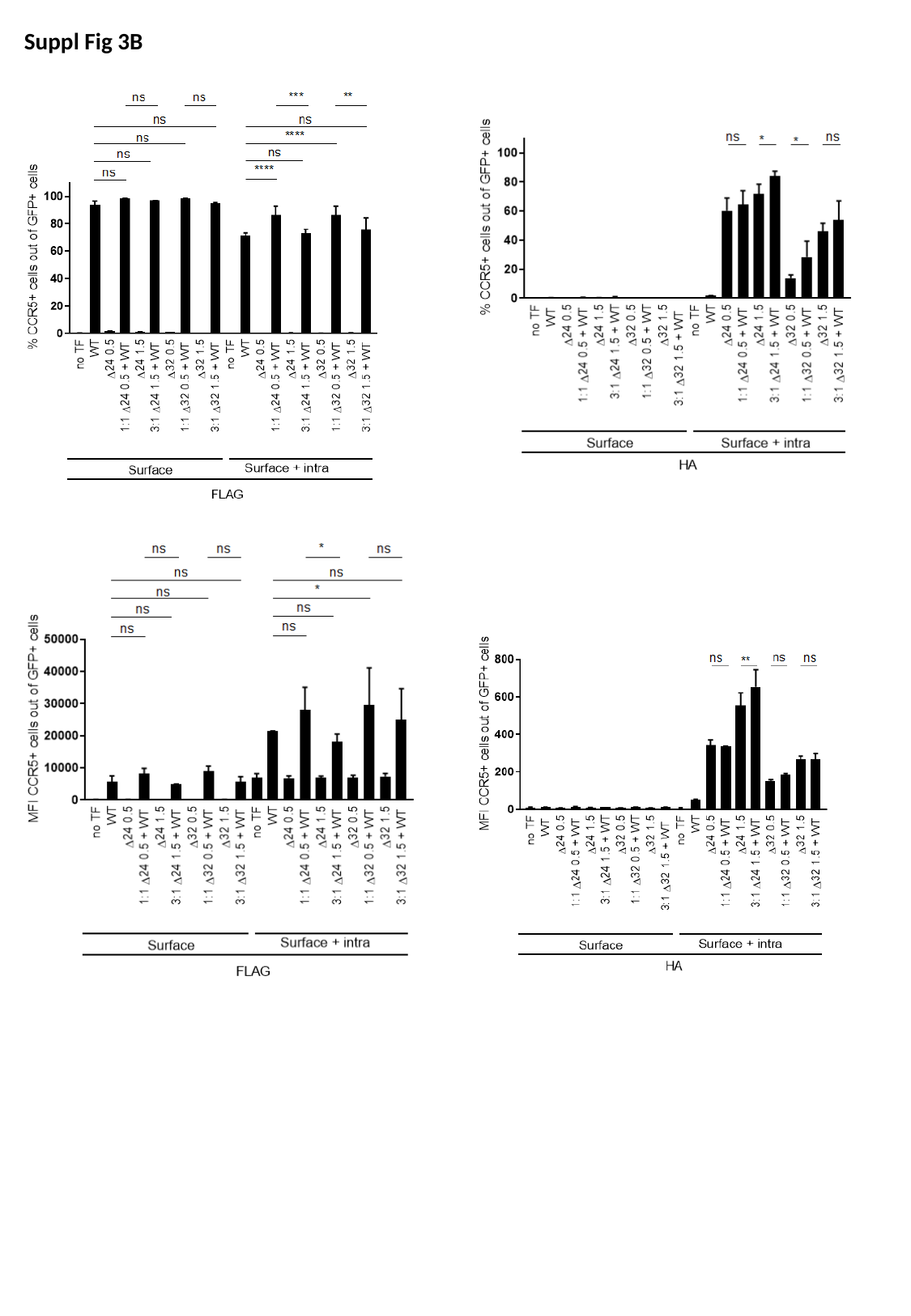

Suppl Fig 3B

## Slide 3
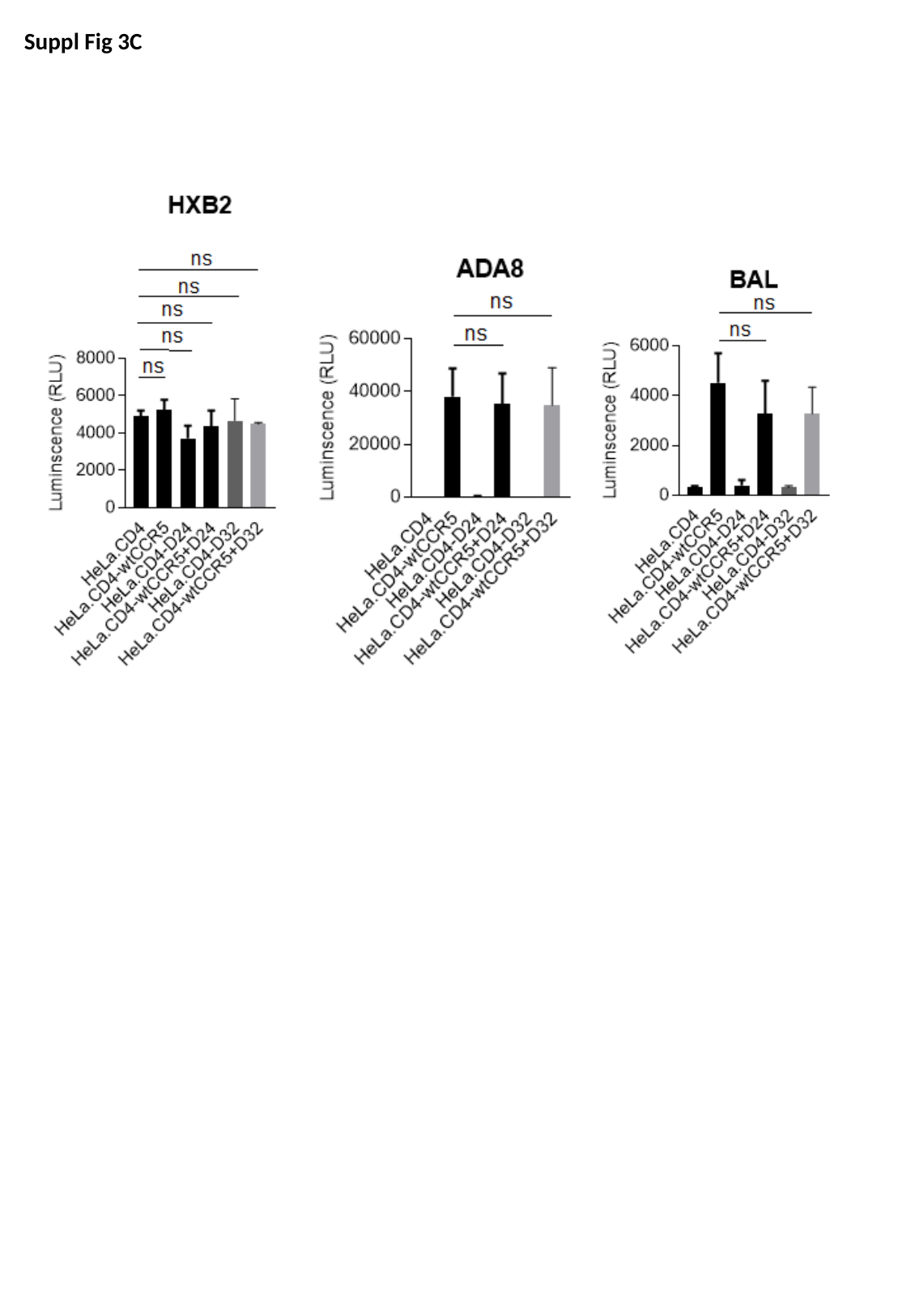

Suppl Fig 3C

Supplement: Supplementary file 3 — Figure S3. hCCR5Δ24 mutant has no transdominant negative effect on wtCCR5 using a 3:1 ratio. (A) HEK‐293T and HeLa‐CD4 cells were transfected with FLAG‐wtCCR5, HA‐hCCRΔ24 and HA‐hCCR5Δ32 alone or cotransfected with FLAG‐wtCCR5 and HA‐hCCRΔ24 or HA‐hCCR5Δ32 at 1:1 or 3:1 ratios. A GFP reporter vector was added to each transfection in order to analyse CCR5 expression in transfected populations. CCR5 surface or surface + intracellular expression was analysed by flow cytometry using anti‐FLAG and anti‐HA mAbs. (A) Reports quantification of the flow cytometry experiments. (B) HeLa‐CD4 cells were transfected with FLAG‐wtCCR5, HA‐hCCRΔ24 alone or cotransfected with FLAG‐wtCCR5 and HA‐hCCRΔ24 at a 3:1 ratio as in (A). (C) Transfected cells were infected with HXB2, ADA8 or BaL pseudovirus expressing a Luciferase reporter gene 48 hours post‐transfection. HIV‐1 infection was quantified by measuring Luciferase‐dependent luminescence. Statistical significance was considered when p ≤ 0.05 (****p ≤ 0.0001, ***p ≤ 0.001, **p ≤ 0.01, *p ≤ 0.05; N = 3 independent experiments). Error bars denote mean ± SD. [file JIA2-22-e25384-s003.pptx]
